# Clinical efficacy of the enzyme replacement therapy in patients with late-onset Pompe disease: a systematic review and a meta-analysis

**DOI:** 10.1007/s00415-021-10526-5

**Published:** 2021-04-13

**Authors:** Berli Sarah, Brandi Giovanna, Keller Emanuela, Najia Nadi, Vitale Josè, Pagnamenta Alberto

**Affiliations:** 1 Institute for Intensive Care Medicine, University Hospital Zurich, Rämistrasse 100, 8091 Zurich, Switzerland; 2 Department of Neurosurgery and Clinical Neuroscience Center, University Hospital and University of Zurich, Zurich, Switzerland; 3 Intensive Care Unit, Regional Hospital Mendrisio, Ente Ospedaliero Cantonale, Bellinzona, Switzerland; 4 Centro Medico, Mendrisio, Switzerland; 5 Unit of Biostatistics, Ente Ospedaliero Cantonale, Bellinzona, Switzerland; 6 Division of Pneumology, University Hospital of Geneva, Geneva, Switzerland

**Keywords:** Pompe disease, Gycogen storage disease type II, Late-onset Pompe disease, Enzyme replacement therapy, Recombinant human alpha-glucosidase, Systematic review, Meta-analyse

## Abstract

In patients with late-onset Pompe disease (LOPD), the efficacy of the enzyme replacement therapy (ERT) with recombinant human alpha-glucosidase (rhGAA) is difficult to evaluate, due to the clinical heterogeneity and the small sample sizes in published studies. Therefore, we conduct a systematic literature review and meta-analysis of the literature to evaluate the efficacy of ERT in LOPD patients considering the walking distance, respiratory function and muscle strength. Particularly, six-minute walk test (6MWT), forced vital capacity (FVC), medical research council (MRC) grading, quantitative muscle testing (QMT), and quick motor function test (QMFT) were outcomes of interest. Overall, 619 studies were identified in PubMed, EMBASE and by manual search on July 18th, 2020. After an initial assessment, 16 studies were included in the meta-analysis, containing clinical data from 589 patients with LOPD. For the 6MWT, 419 patients were analyzed. Walking distance improved on average, 32.2 m greater during the observed period (*p* = 0.0003), compared to the distance at the baseline. The meta-analysis did not show any improvement in FVC and only a tendency towards better muscle strength after treatment with ERT, but the difference was not statistically significant. In conclusion, the available data showed that ERT has a significant beneficial efficacy in the improvement of walking distance in LOPD patients and a non-significant improvement of muscle strength. No improvement in respiratory capacity was found. More prospective and controlled trials are needed to demonstrate a clear clinical benefit of ERT.

## Introduction

Pompe disease (PD) is a rare, inheritable, multisystemic lysosomal storage disorder caused by a deficiency of acid alpha-glucosidase (GAA), which leads to accumulation of glycogen within lysosomes, especially in the heart, skeletal and smooth muscles, and in the nervous system [1, 2]. Two forms of PD—infantile-onset (IOPD) and late-onset (LOPD)—are classically recognized. Depending on the age of symptoms’ onset, LOPD is sub-classified in a juvenile (JOPD) and in an adult form [3, 4]. IOPD is the most severe form and is characterized by cardiomegaly and generalized muscle weakness presenting during the first few months of life. LOPD, indeed, is clinically more heterogeneous [5], involving primarily skeletal and respiratory muscles leading to proximal weakness and respiratory insufficiency typically without cardiac manifestations [2, 5]. Respiratory failure represents the leading cause of death in LOPD patients [1, 6, 7].

In 2006 the first specific enzyme replacement therapy (ERT) with recombinant human GAA (rhGAA) was approved for PD treatment in Europe and the US [8] with the recommended dose of 20 mg/kg via IV infusion biweekly [6]. In IOPD patients, previous ERT studies already demonstrated prolonged long-term survival, improved cardiac function, and a decreased need for respiratory support [9]. In patients with LOPD, on the other hand, it is difficult to assess the general long-term effects of ERT on disease progression from individual studies due to a wide range of phenotypic presentations, the very low prevalence of the disease, and the small sample sizes in published studies. Systematic literature reviews assessing the impact of ERT in LOPD patients were previously conducted [10–12]. Toscano et al. found that at least two-thirds of treated patients showed improvement or stabilization of muscular and respiratory function after initiation of ERT [10]*.* Schoser et al. reported in a systematic review and meta-analysis a nearly five-fold lower mortality rate in treated patients than untreated patients and an improved respiratory function within the first few months followed by a slow return to baseline and an improved muscular function, which remains sustained over time [11]. By the consensus meetings of the European Pompe Consortium, a systematic review with a narrative synthesis due to the expected heterogeneity was undertaken showing a beneficial effect of ERT at the group level but with varied treatment effects at the individual level [12]. For JOPD patients only, a systematic literature review without statistical synthesis due to a low quality of original articles suggested a short-term benefit from ERT through improved muscle strength and reduced need for assisted ventilation [13].

The present work provides an important update on the current evidence on the clinical efficacy of ERT in patients with LOPD with regard to motor performance, respiratory function, and muscle strength compared to their baseline values. A systematic literature review with meta-analysis was conducted to assess the efficacy of treating LOPD with ERT on six-minute walk test (6MWT), forced vital capacity (FVC), and muscle strength measured with medical research council (MRC), quantitative muscle testing (QMT) and quick motor function test (QMFT).

## Material and methods

PRISMA guidelines (Preferred Reporting of Items in Systematic Reviews and Meta-analyses) [14] were employed to guide review processes.

This systematic literature review was registered at the international prospective register of systematic reviews (PROSPERO; registration number: CRD42020182462).

### Search strategy

Studies were identified with the MEDLINE (1966 to July 18th, 2020) and EMBASE (1980 to July 18th 2020) electronic databases. The search strategy was developed with the following keywords: *Pompe disease*, *alpha glucosidase deficiency*, *acid maltase deficiency, glycogen storage disease type 2, enzymatic replacement therapy, enzyme replacement therapy*, *forced vital capacity*, *FVC*, *respiratory function*, *six-minute walk test*, *6MWT, medical research council*, *MRC*, *quantitative muscle testing*, *QMT*, *quick motor function test*, *QMFT*. The electronic search was supplemented by a manual search of reference lists and recent reviews, and by reviewing abstract books from the congress abstracts from the annual meetings.

Two reviewers (SB and GB) independently screened titles and abstracts and duplicates. Secondly, they screened the corresponding publications in full text to assess if the studies met the inclusion criteria. All disagreements were resolved by discussion between the two reviewers. All persisting disagreements were reviewed and resolved by a third conciliator (NN). The final inclusion of studies was based on the agreement of all reviewers.

### Inclusion and exclusion criteria

The study selection criteria are presented in Table 1 using the PICOS (Population, Interventions, Comparisons, Outcomes, and Study design) acronym.

**Fig. 1:**
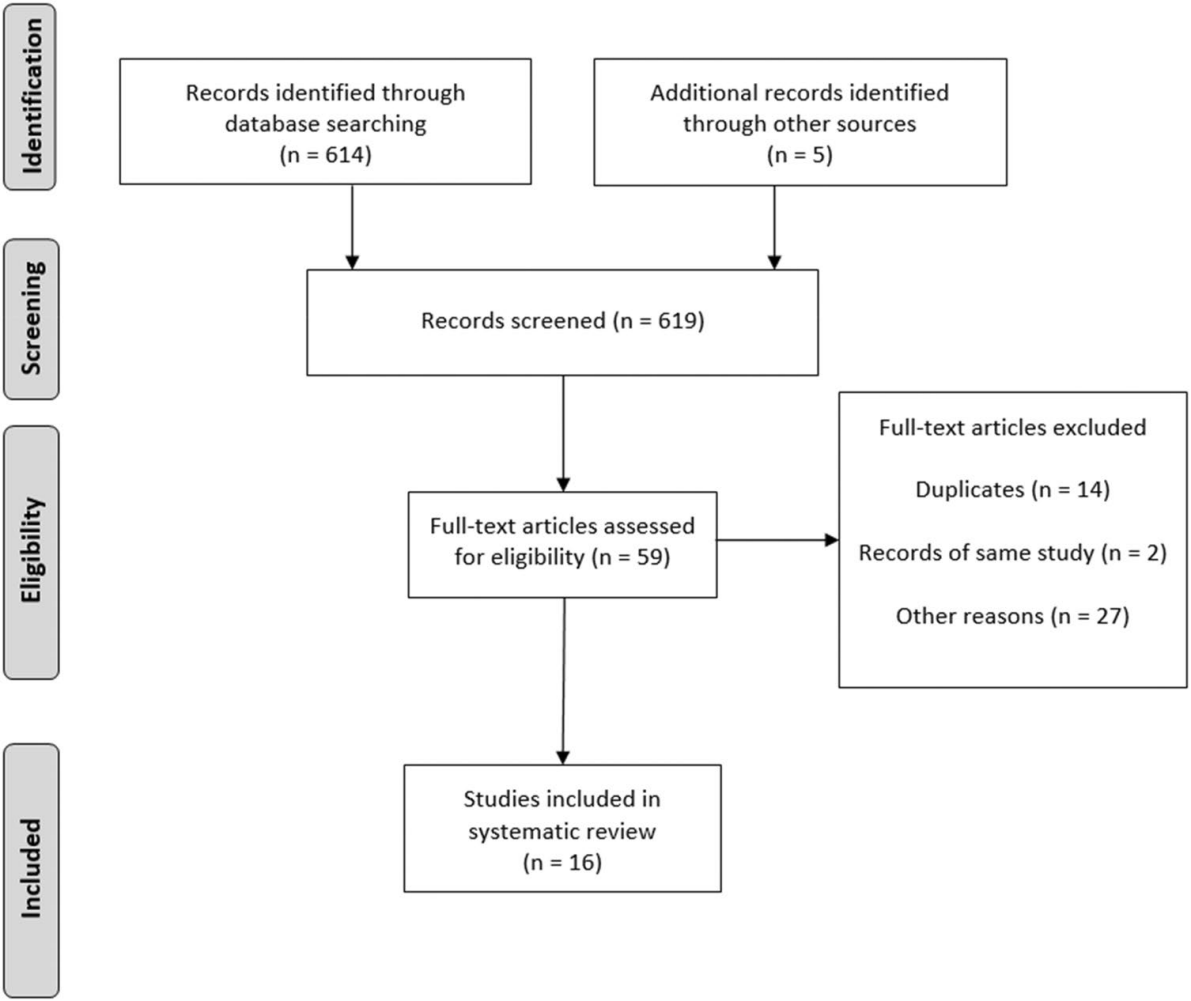
Study selection Flow diagram

All identified findings were reported using a flow chart according to PRISMA statement (Fig. 1).

Randomized controlled trials (RCTs), prospective observational studies, and retrospective studies with more than ten patients that evaluated rhGAA effects in patients with LOPD were eligible.

Case reports, case series with less than ten subjects, studies in patients with IOPD, experimental studies, abstracts, studies without baseline data or control group, and reports with no values in the results were excluded. The search was limited to articles written in English.

### Data extraction

The extracted data were: trial’s characteristics (first author, publication year), kind of study, treatment doses, disease duration at the start of ERT (from symptom onset), control group, selected outcome results baseline and after treatment begin with ERT (value, difference, P-value) and follow-up duration with ERT. Information was summarized using a pre-defined Excel Table.

Outcomes of interest were: motor performance, as assessed by the 6MWT; respiratory function, by considering the FVC; and muscle strength, as tested with the MRC grading scale, QMT and QMFT.

### Statistical analysis

For each specific outcome (motor performance, respiratory function and muscle strength) the effect estimate and its corresponding 95%-confidence interval (95%-CI) were calculated for each study separately according to the inverse-variance fixed-effect method [15]. Each trial-specific effect size was subsequently combined across studies to calculate summary estimates and presented as a forest plot. Effect size on motor function was assessed on mean difference in 6MWT, effect size on respiratory function was evaluated on mean difference in FVC, whereas effect size on muscle strength was assessed on the standardized mean difference. The test statistic *Q* was applied to assess the statistical evidence of heterogeneity. Due to the conservative nature of the test a cut-off of *p* < 0.1 was used. For the estimation of the degree of heterogeneity *I*^2^ statistic was used. The presence of publication bias was explored with a funnel plot. All analyses were performed using RevMan 5.3 (Review Manager (RevMan) [Computer program]. Version 5.3. Copenhagen: The Nordic Cochrane Centre, The Cochrane Collaboration, 2014).

## Results

The systematic search identified 614 studies in the databases PubMed and EMBASE. Through manual search five additional papers were identified. Overall, 619 abstracts were considered as potentially eligible. After screening based on the inclusion criteria, 59 were selected for full-text review, as shown in the flow diagram of Fig. 1. Finally, a total of 16 studies were included in the meta-analysis, containing clinical data from 589 patients with LOPD [1, 7, 16–29].

The list and characteristics of the included papers are presented in Table 2: 6-min walk test (6MWT); forced vital capacity (FVC); and muscle strength (MRC), (QMT) and (QMFT).

The included studies were published between 2010 and 2020.

### Motor performance: 6-min walk test

**Fig. 2:**
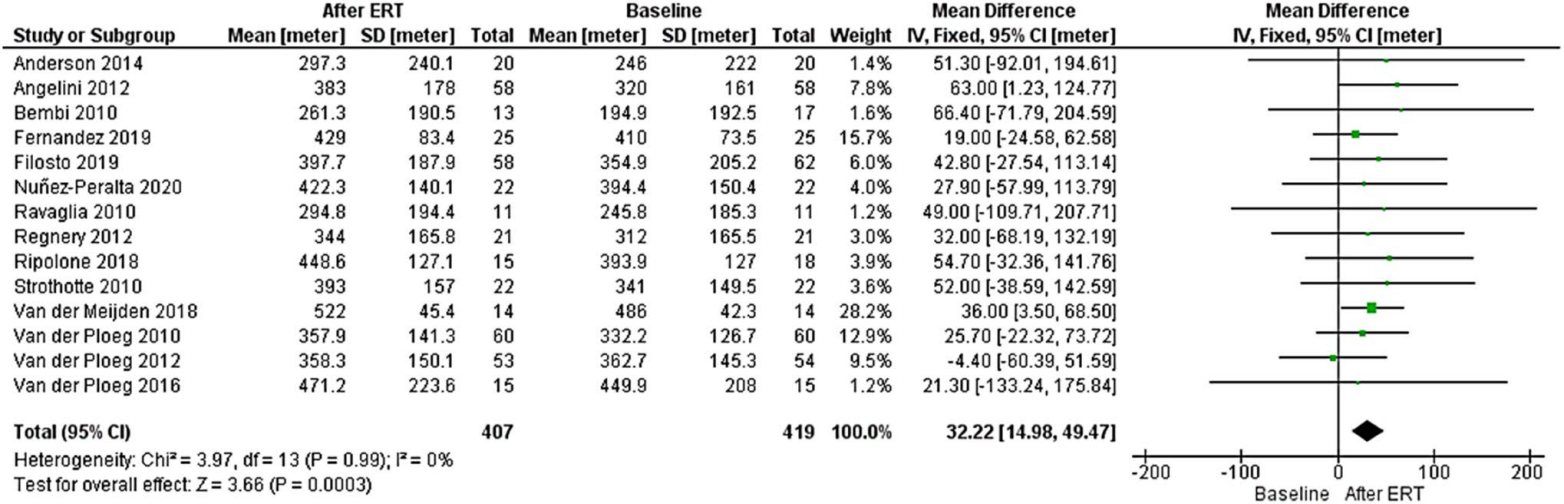
Forest plot of effect of enzyme replacement therapy on motor performance, as assessed by 6-min-walk test

Fourteen studies, including 419 patients with LOPD (of those, 407 were treated with rhGAA) reported results concerning 6MWT as shown in Fig. 2 [1, 7, 16–19, 21–23, 25–29]. Follow-up time varied from 6 months to 7 years.

Forest plot of stratified analysis demonstrated that after 18.75 months, patients on rhGAA showed significant improvements in walking distance that were, on average, approximately 32.22 m (95%-CI 14.98–49.47; *p* = 0.0003) greater during the observed period, compared to the distance at baseline time.

**Fig. 3:**
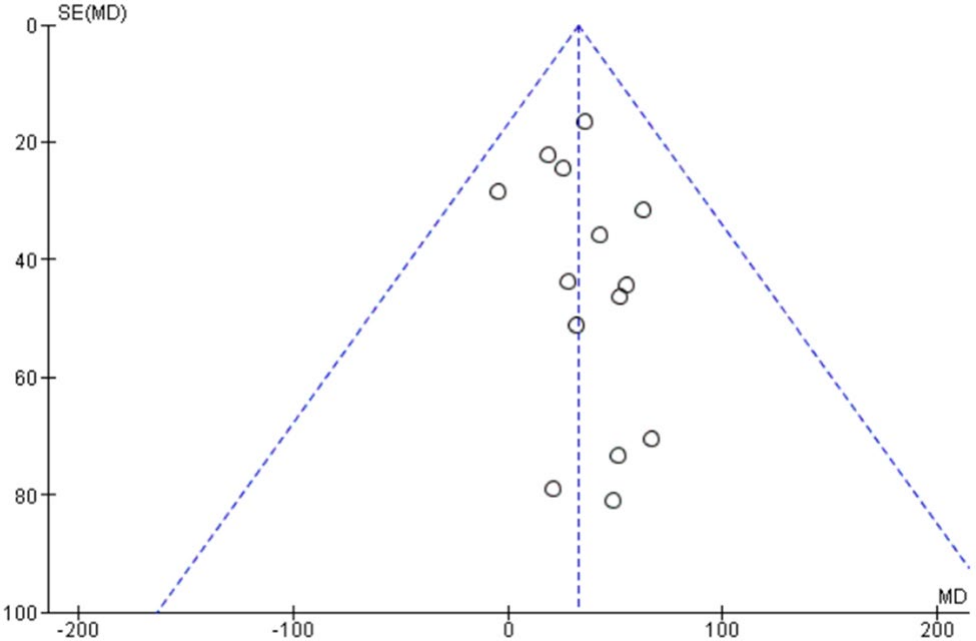
Funnel plot of comparison of 6-min-walk test

Assessment of publication bias using a funnel plot indicated symmetry as shown in Fig. 3.

### Respiratory function: forced vital capacity

**Fig. 4:**
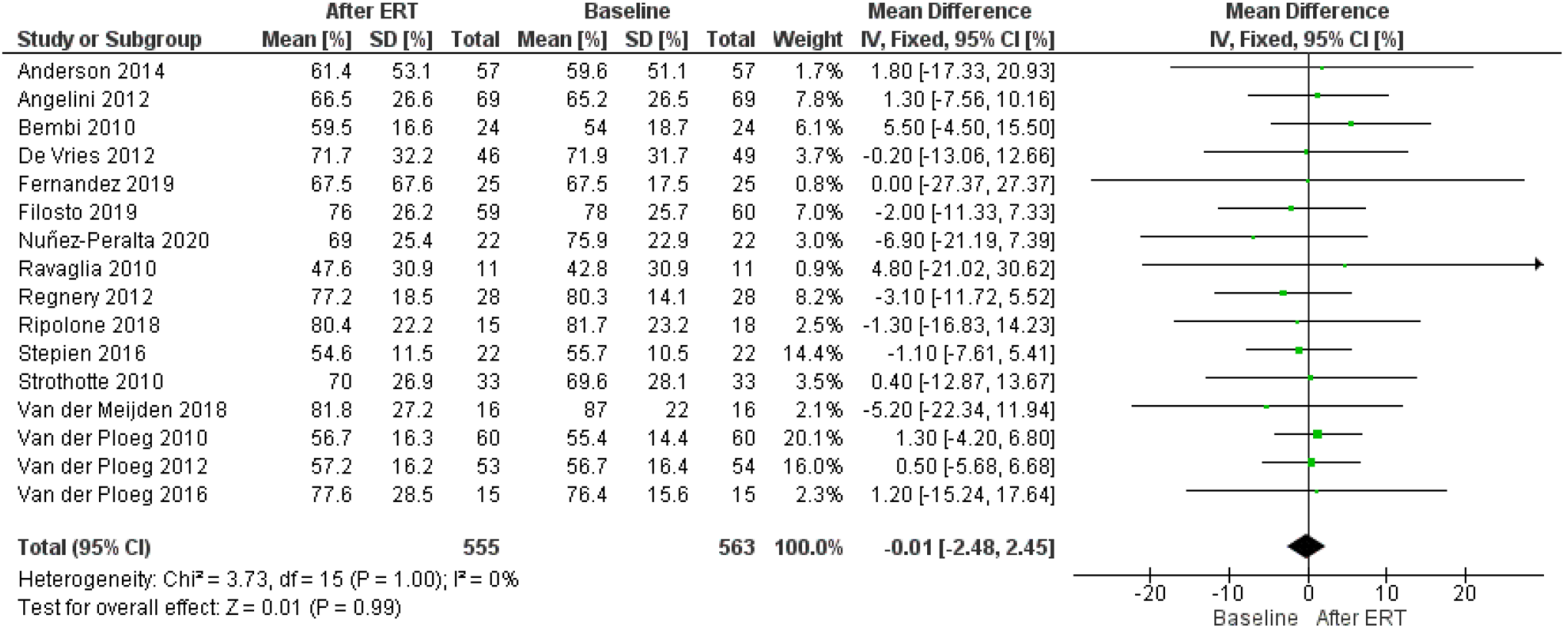
Forest plot of effect of enzyme replacement therapy on respiratory function, as assessed by forced vital capacity

Sixteen studies, including 563 patients with LOPD (of those, 555 were treated with rhGAA) reported results concerning (FVC) as shown in Fig. 4 [1, 7, 16–29].

Follow-up time varied from 6 months to 7 years.

Forest plot of stratified analysis demonstrated that after 20.75 months, patients on rhGAA did not show significant changes. FVC remained stable with approximately − 0.01% (95%-CI − 2.48–2.45; *p* = 0.99) during the observed period, compared to the FVC at the baseline time.

**Fig. 5:**
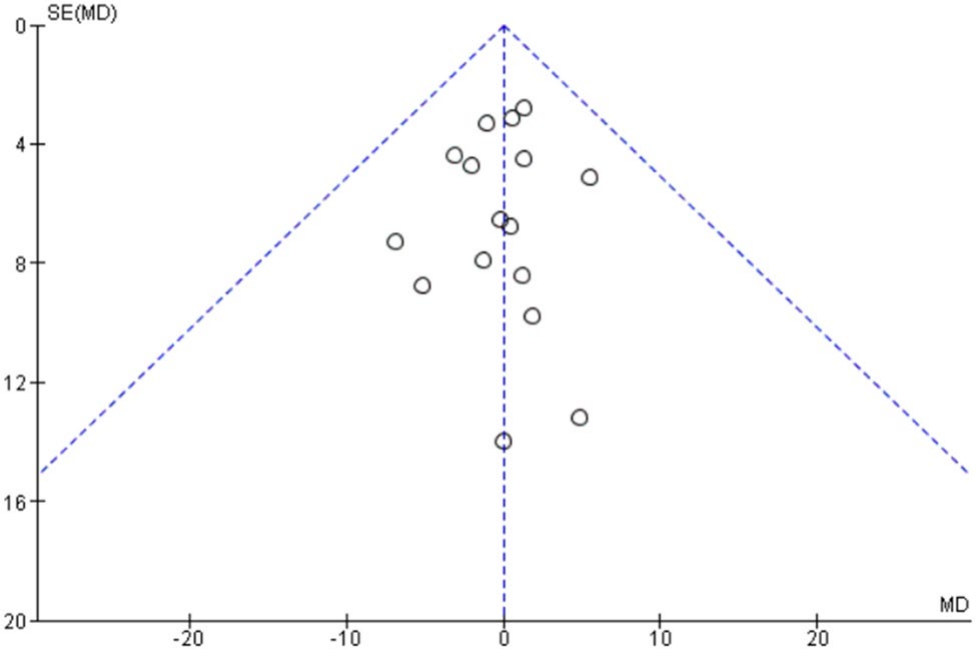
Funnel plot of comparison of forced vital capacity

Assessment of publication bias using a funnel plot indicated symmetry as shown in Fig. 5.

### Muscle strength: medical research council grading scale, quantitative muscles testing and quick motor function test

**Fig. 6:**
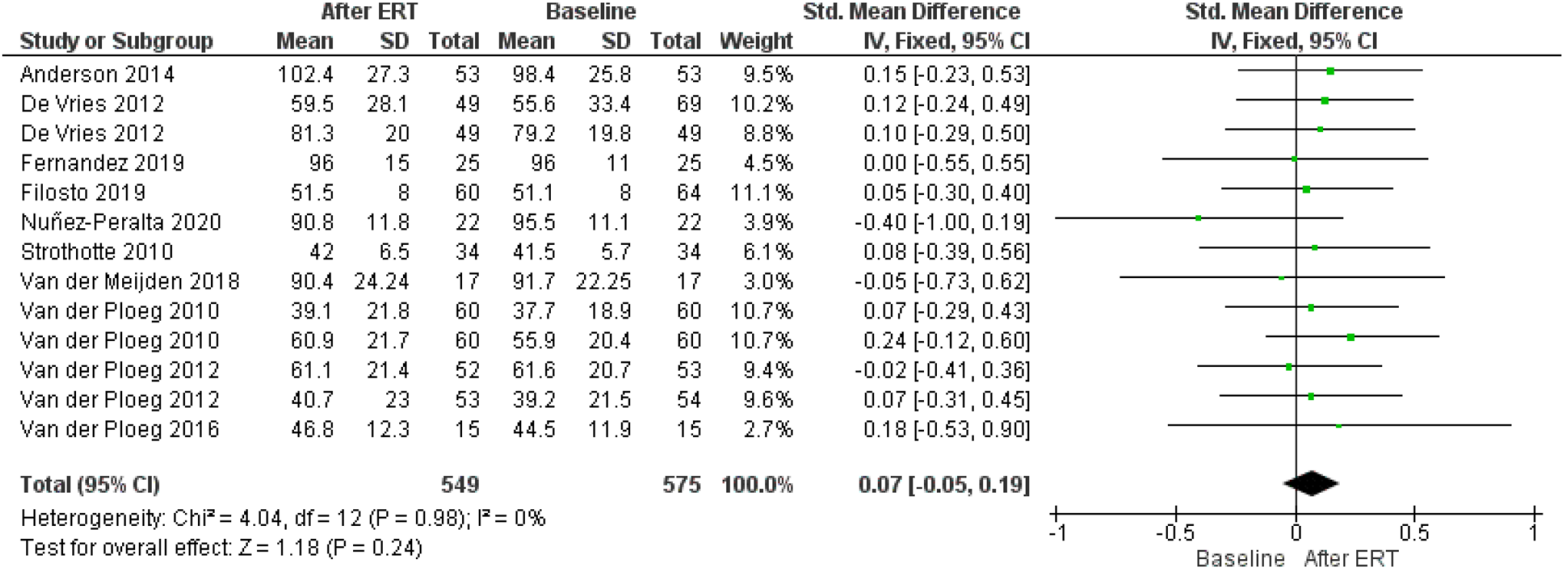
Forest plot of effect of enzyme replacement therapy on muscle strength, as assessed by medical research council grading scale, quantitative muscles testing and quick motor function test

Ten studies, including 413 patients with LOPD (of those, 388 were treated with rhGAA) reported 575 measurements of muscle strength at baselines and 549 measurements after ERT assessed with MRC, QMT or QMFT as shown in Fig. 6 [1, 7, 16, 17, 19–24, 26–29].

Follow-up time varied from 6 months to 7 years.

Forest plot of stratified analysis demonstrated that after 19.5 months, patients on rhGAA did not show significant improvements in muscle strength even though the average shows an increase of approximately 0.07 points (95%-CI − 0.05–0.19; *p* = 0.24) during the observed period, compared to muscle strength at baseline.

**Fig. 7:**
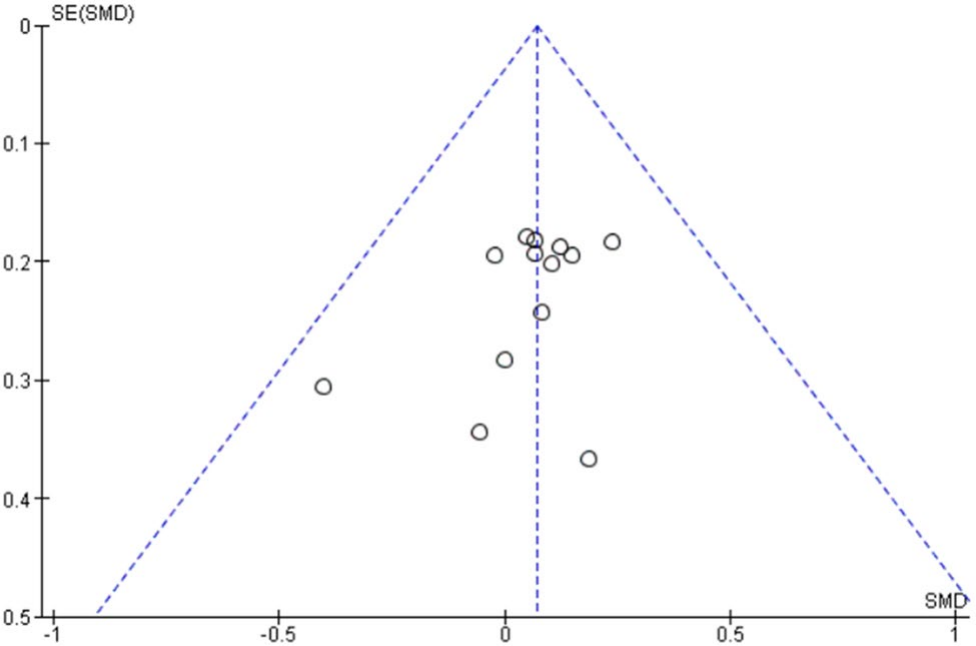
Funnel plot of comparison of medical research council grading scale, quantitative muscles testing and quick motor function test

Assessment of publication bias using a funnel plot indicated symmetry as shown in Fig. 7.

Two of the included studies [1, 21] reported muscle strength results in two groups as composite QMT leg and arm scores (i.e., the average of the percentage of predicted scores for bilateral knee flexors and extensors and bilateral elbow flexors and extensors). To optimize the number of results for our search, both determinations were included for the analysis, which represents the same population.

## Discussion

### Main findings

Fourteen years after ERT became available, we conducted this systematic review to update the previous findings and to perform a meta-analysis on the efficacy of ERT in patients with LOPD. Outcomes of interest were walking distance, respiratory function, and muscle strength after begin of ERT compared to their baseline values. As the main result, we found that in patients with LOPD after ERT the walking distance significantly improved, however, the respiratory function and the muscle strength remained unchanged.

### Results in context

Güngör and co-workers reported a survival benefit under ERT [30]. In a recent review with 368 patients, ERT was associated with improved or stabilized muscular and respiratory functions [10]. More recently, a systematic review on survival and muscular function reported a nearly five-fold lower mortality rate. Furthermore, 6MWT improved over the first 20 months of therapy followed by a stabilization [11].

Regarding the FVC, Schoser and co-worked reported a rapid improvement of the respiratory function under ERT, followed by a slow regression to baseline over three-years period, while among untreated patients the respiratory function declined over time [11]. Similarly, our meta-analysis showed that the FVC of patients treated with ERT did not improve beyond 20.75 months. However, at an individual level, the response to treatment varied. The reasons for this variation in clinical response need further investigation, especially since respiratory failure represents the leading cause of death in patients with LOPD [1, 6, 7]. Phenotypic, and genotypic factors associated with LOPD might likely play a role. In addition, a diagnostic delay that may postpone an earlier begin of ERT might influence the treatment response. Since the deterioration of lung function is the major cause of mortality in patients with LOPD, even if ERT does not produce a significant improvement in FVC, it would have a substantial impact on patient survival, by preventing or slowing down the decline of the respiratory function.

In summary, the results of our meta-analysis confirm the findings of the previous systematic review. Unlike from the previous studies, however, we investigated a third outcome, the muscle strength. We decided to include also this endpoint because all together, 6-MWT, FVC, and muscle strength represent the first recommended functional assessment by the Swiss Guidelines of Pompe disease [31], in monitoring the patients, and are the same outcomes recommended by the European Pompe Consortium [12]. In the analysis, we included studies that evaluated the muscle strength using the MRC grading scale, the QMT and the QMFT, which are the most used tools to evaluate muscle strength in Europe [32] and they also were already used in previous reviews on patients with JOPD [13]. Additionally, we performed a meta-analysis to investigate the efficacy of ERT on muscle strength in patients with LOPD. In the identified studies, referring to 413 patients treated with ERT, the muscle strength did not increase significantly, despite a trend towards improvement of muscle strength was observed.

### Implications for research

Phenotypic and genotypic factors might have a role in the prediction of the therapeutic response to ERT in patients with LOPD. In addition, the development of antibodies against ERT, which may reduce its effect could influence the efficacy of the therapy [33, 34]. Further studies are needed to better identify these factors and, hence, patients who might benefit from the therapy. In fact, knowing which patients have a better response would allow a more efficient patient prioritizing and an optimizing of resources. Especially in countries with limited resources this might be of concern [35].

### Implications for practice

Some studies have suggested that early initiation of ERT may improve the clinical response to the treatment [1, 17, 36]. Given that, the American Association of Neuromuscular and Electrodiagnostic Medicine [37] recommends starting ERT in all symptomatic patients with a confirmed diagnosis. Similarly, the European Pompe Consortium [12] recommends initiating ERT in symptomatic patients who agree to regular treatment and monitoring, have residual skeletal and respiratory muscle function, and do not have another life-threatening illness in an advanced stage. However, to better identify the predictors of the therapeutic response, it is crucial to continue to collect data on cohorts of patients with LOPD. Furthermore, due to the growing interest for the use of neo-GAA as well as ERT combined with a chaperon in patients with LOPD, further studies are needed to verify the effectiveness of these new treatments [38].

### Strengths/Limitations

Strengths of this review are that several new publications have been added compared to the previous systematic reviews, and at the same time, the overlap of patients between studies was taken into account. Furthermore, this work represents one of the few systematic reviews including a meta-analytic design referring specifically to patients with LOPD. Furthermore, muscle strength was included as an outcome of interest.

This review has also limitations. First, only a relatively small number of studies with a small number of patients have been included. However, this is not surprising given how rare LOPD is. To limit this bias, we included in the analysis only studies with more than 10 patients and we excluded clinical cases. Second, considering the variety of settings and designs of the included studies, results are susceptible to selection bias. Third, we limited the evaluation of the efficacy of ERT in patients with LOPD on 6MWT, FVC, and muscle strength. Some further outcomes, such as clinical improvement perceived by patients, measurements specifically developed to evaluate LOPD or at least more specific to neuromuscular disease, should also be considered (39). Finally, outcomes of interest in the included studies were evaluated at different follow-up times, from 6 months to 7 years after initiation of ERT, limiting the generalization of our findings.

## Conclusions

In conclusion, this study updates previous literature reviews on the efficacy of ERT in patients with LOPD. It shows that in patients with LOPD treated with ERT, the walking distance significantly improved while the muscle strength only showed a non-significant improvement, though the respiratory function did not change. Only a few studies had an adequate methodological quality to be included in our meta-analysis; therefore, new clinical trials are necessary to obtain more robust results about the clear clinical benefit of ERT.

**Table 1:** Inclusion criteria: Scope of the literature review in PICOS form

Criteria	Definition
Population	Patients with LOPD
Interventions	Recombinant human GAA 20 mg/kg every two weeks
Comparison	Patients baseline values
Outcomes	6-min walk test (6MWT) Forced vital capacity (FVC) Muscle strength: medical research council (MRC) grading, quantitative muscle testing (QMT), quick motor function test (QMFT)
Study Design	RCTs Open-label extension phases of included RCTs Single-arm trials Prospective observational studies Retrospective studies with more than 10 patients

**Table 2:** Characteristics of the included papers

Primary Author (Year)	*N*	6MWT Mean (meter)				Muscle Strength Mean (MRC:*/QMT:°/QMFT:#)						FVC Mean (%)			
		Baseline		After ERT		Baseline				After ERT		Baseline		After ERT	
Ravaglia et al. [16]	11	245.8		294.8								42.8		47.6	
Strothotte et al. [17]		*N* = 22	341	*N* = 22	393	*N* = 34		41.5*		*N* = 34	42*	*N* = 33	69.6	*N* = 33	70
Bembi et al. [18]		*N* = 17	194.9	*N* = 13	261.3							*N* = 24	54	*N* = 24	59.5
Van der Ploeg et al. [1]	60	332.2		357.9		Arm		55.9°		60.9°		55.4		56.7	
						Leg		37.7°		39.1°					
Angelini et al. [19]		*N* = 58	320	*N* = 58	383							*N* = 69	65.2	*N* = 69	66.5
De Vries et al. [20]						*N* = 49		79.2*		*N* = 49	81.3*	*N* = 49	71.9	*N* = 46	71.7
						*N* = 69		55.6#		*N* = 49	59.5#				
Van der Ploeg et al. [21]		*N* = 54	362.7	*N* = 53	358.3	Arm	*N* = 53		61.6°	*N* = 52	61.1°	*N* = 54	56.7	*N* = 53	57.2
						Leg	*N* = 54		39.2°	*N* = 53	40.7°				
Regnery et al. [22]		*N* = 21	312	*N* = 21	344							*N* = 28	80.3	*N* = 28	77.2
Anderson et al. [23]		*N* = 20	246	*N* = 20	297.3	*N* = 53		98.4*		*N* = 53	102.4*	*N* = 57	59.6	*N* = 57	61.4
Van der Ploeg et al. [7]	15	449.9		471.2		44.5#				46.8#		76.4		77.6	
Stepien et al. [24]	22											55.7		54.6	
Ripolone et al. [25]		*N* = 18	393.9	*N* = 15	448.6							*N* = 18	81.7	*N* = 15	80.4
Van der Meijden et al. [26]		*N* = 14	486	*N* = 14	522	*N* = 17		91,7*		*N* = 17	90,4*	*N* = 16	87	*N* = 16	81,8
Ferndandez et al. [27]	25	410		429		96*				96*		67,5		67,5	
Filosto et al. [28]		*N* = 62	354.9	*N* = 58	397.7	*N* = 64		51.1*		*N* = 60	51.5*	*N* = 60	78	*N* = 59	76
Nuñez-Peralta et al. [29]	22	394.4		422.3		95.5*				90.8*		75.9		69	
